# Asymptomatic Subglottic Stenosis Discovered During Anesthesia Induction and Not Predicted by Preoperative Evaluation: A Case Report

**DOI:** 10.7759/cureus.59543

**Published:** 2024-05-02

**Authors:** Ayaka Hibino, Akinobu Hibino, Hironobu Nishimaki, Sadahei Denda

**Affiliations:** 1 Anesthesiology, Niigata University Medical and Dental Hospital, Niigata, JPN; 2 Anesthesiology and Pain Clinic Surgery, Niigata City General Hospital, Niigata, JPN

**Keywords:** general anesthesia, laryngeal mask, supraglottic apparatus, difficult intubation, tracheal intubate, subglottic stenosis

## Abstract

Subglottic stenosis (SGS) can be asymptomatic in cases with slow-growing granulomas. In this study, we report a case of SGS discovered during tracheal intubation for anesthesia induction. A 74-year-old woman was scheduled for surgery under general anesthesia for a left humeral fracture. Resistance was observed when the tracheal tube passed through the glottis, stopping the tube from advancing. We placed a laryngeal mask (LMA) to secure her airway and examined it using a bronchial fiber to detect circumferential stenosis of the subglottis due to granulation. The airway was secured using an LMA instead of intubation, and the patient was successfully managed under anesthesia. Asymptomatic SGS is difficult to detect preoperatively, and anesthesiologists may encounter unexpected intubation issues. LMA is an important tool for an effective strategy to manage intubation difficulties.

## Introduction

Subglottic stenosis (SGS) can be caused by trauma to the larynx, long-term tracheal tube placement, burns, infectious diseases including tuberculosis, and systemic diseases such as Wegener's granuloma [1]. SGS can present with respiratory symptoms, including wheezing and dyspnea; however, in cases with slow-growing granulomas, subjective symptoms may be limited [2]. In this study, we report the case of a patient with SGS that was not predicted during preoperative evaluation and was first detected during intubation complications encountered during general anesthesia induction.

This article was previously presented as a meeting abstract at the 62nd Joint Meeting of the Kanto Koshinetsu and Tokyo Sections of the Japanese Society of Anesthesiologists (2022, Tokyo). The patient provided written informed consent for the publication of this case report.

## Case presentation

In this study, we present the case of a 74-year-old woman (height, 148 cm; weight, 79.5 kg) who presented to our hospital with a left humeral diaphysis fracture sustained during a fall at home. The patient was then scheduled for intramedullary nail fixation under general anesthesia the following day. Her surgical history included cesarean sections at 34 and 36 years of age and bilateral knee joint replacement at 70 years of age. Underlying medical conditions included hypertension, type 2 diabetes mellitus, and bronchial asthma. Preoperative electrocardiography and chest radiography revealed no abnormal findings. No respiratory function tests were performed. HbA1c was 7.6%.

**Figure 1:**
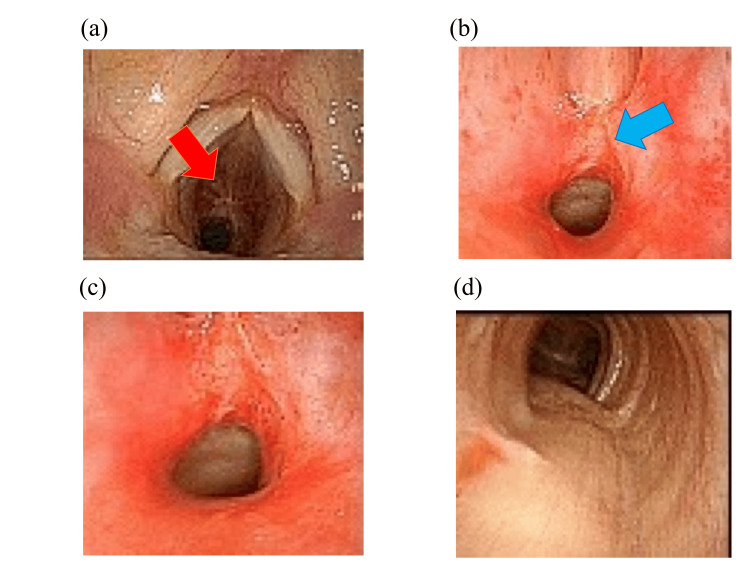
Images of the trachea around the site of subglottic stenosis and peripherally observed by fiberoptic bronchoscopy. (a) Membranous structures observed at the level of the glottis. (b) Scar tissue just below the vocal cords. (c) Tracheal stenosis. (d) Trachea at the peripheral side from the site of tracheal stenosis.

For anesthesia, the patient was pre-oxygenated with 5 L/min of oxygen for approximately five minutes, followed by rapid induction with propofol (80 mg), rocuronium (50 mg), and remifentanil (0.25 mcg/kg/min). Subsequently, 6% desflurane was administered after confirming the disappearance of the eyelash reflex. Mask ventilation was easy, and fully opened vocal cords were observed on larynx expansion using McGRATH®. Intubation was attempted using a spiral endotracheal tube with an internal diameter (ID) of 7.0 mm (ShileyTM TaperGuardTM Tracheal Tube, Covidien Japan, Japan). However, as the tracheal tube tip passed through the vocal cords, resistance was observed and the tube did not advance further. Therefore, we assumed that the tracheal run may have varied from the insertion angle and attempted to re-insert the tube while rotating it; however, no change in the resistance was observed from the tube tip and intubation was not possible. Subsequently, the glottis area was observed closely on the McGRATH® monitor, and a structure was observed just below the glottis (Fig. 1a). Although the structure seemed to obstruct the intubation, we predicted that the airway could be secured with a laryngeal mask (LMA) (ProSealTM, SENKO MEDICAL INSTRUMENT, Japan), as mask ventilation was easy. Therefore, we temporarily interrupted intubation and secured the airway using the LMA. The ventilator was configured with a ventilation volume of 500 mL, 10 breaths per cycle, and a positive end-expiratory pressure of 6 cmH_2_O in the VC mode. Although the airway pressure was slightly higher (25 cmH_2_O), patient ventilation was confirmed without any problems. We examined the subglottis more closely using fiberoptic bronchoscopy (FOB) (New C-MAC® FIVE S, KARL STORZ SE & Co. KG, Germany, 3.5 mm in outer diameter).

We suggest that the structures immediately below the glottis may have been formed by the cicatricial tissue (Fig. 1b). Furthermore, FOB revealed that the tracheal wall was narrow and covered with granulation tissue (Fig. 1c). The FOB passed through the stenotic area, and the tracheal structure was confirmed to be normal beyond the stenotic area (Fig. 1d). These findings are presented in Figure 1a-c. SGS was diagnosed and an otorhinolaryngologist was consulted to assess the need for an emergency tracheotomy. As the patient was asymptomatic and the airway was secured by an LMA with easy mask ventilation, emergency tracheotomy was not necessary. Intraoperative anesthesia was maintained using oxygen (1 L/min), air (1.5 L/min), desflurane (4 %), and remifentanil (0.1 mcg/kg/min). The surgeon suggested that the patient could be operated on at a more moderate angle than the usual beach chair position as the LMA airway was secured without any problems in this position. The surgery was completed without any complications.

**Figure 2:**
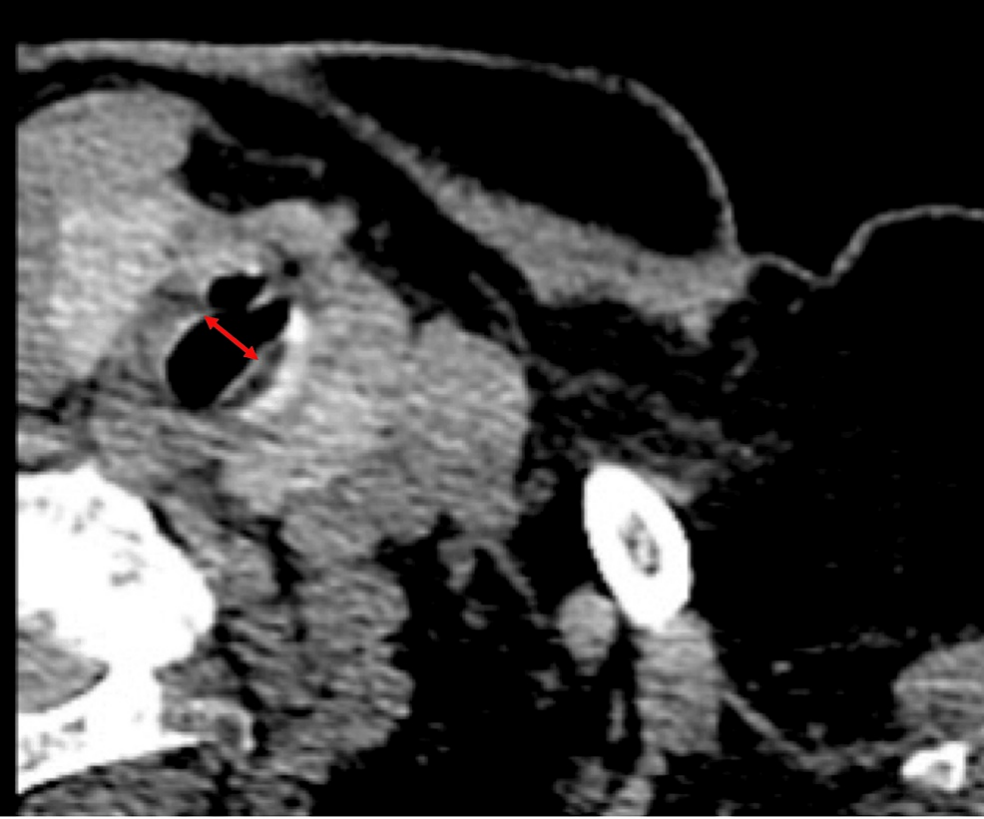
SGS on the preoperative CT image. The red arrow in the CT image shows the site of the SGS. The diameter of the SGS was approximately 7 mm. SGS: subglottic stenosis, CT: computed tomography

Preoperative computed tomography (CT) scans revealed that the diameter of the trachea in the relevant area was approximately 7 mm (Fig. 2). Furthermore, postoperative re-examination of the patient’s medical history revealed that emergency general anesthesia was administered for the first cesarean section at 34 years of age due to fetal distress. Cesarean section at 36 years of age was performed under spinal subarachnoid anesthesia, and bilateral knee arthroplasty at 70 years of age was performed under general anesthesia, although the airway was secured with an LMA, according to her previous anesthesia records. The only time she was intubated was for an emergency cesarean section at 34 years of age.

Postoperatively, the patient visited an otorhinolaryngologist. The SGS in this case was most likely caused by a fine scar on the tracheal wall below the glottis during a previous tracheal intubation, leading to the gradual formation of a scar over many years, resulting in stenosis. Although the stenosis was moderate and amenable to surgical treatment, the scar was considered to have existed for many years, and the patient was asymptomatic, requiring no aggressive intervention unless tracheal intubation was necessary.

## Discussion

SGS can be induced by both congenital and acquired causes. Acquired causes include laryngeal trauma, long-term tracheal intubation or tracheostomy, burns, infectious diseases including tuberculosis, systemic diseases including Wegener’s granuloma and sarcoidosis, and malignant neoplasms [1]. Specifically, long-term tracheal intubation and tracheostomy account for 90% of the acquired SGS cases [3].

SGS symptoms include dyspnea, wheezing, cough, hoarseness, and cyanosis; however, in cases with acquired and slow-forming SGS, symptoms or asthma diagnosis are often absent [2]. Patients do not experience dyspnea and wheezing until their tracheal diameter reduces by approximately 50% of the original size. Furthermore, these symptoms do not become apparent until the tracheal diameter reduces by approximately ≥75% [4]. The average tracheal diameter in women aged 70-79 years is reported to be 16.4 ± 2.4 mm [5]. As the tracheal diameter in Japanese adults is approximately 2 mm narrower than that in Western adults, the tracheal stenosis in this case was approximately 47% of the tracheal diameter. Therefore, the patient did not present with symptoms, such as wheezing and dyspnea.

No preoperative respiratory function tests were performed. SGS can be diagnosed based on extra-thoracic airway obstruction predicted by an expiratory disproportion index (the ratio of FEV1 to peak expiratory flow rate) > 0.5 [6]. In this case, a respiratory function test could have predicted the possibility of upper airway stenosis, and CT images could have helped in the early diagnosis of SGS, considering the patient history.

In many cases of emergency surgery, a CT scan would be taken to evaluate the surgical site. When CT images of the cervicothoracic region are also taken, the anesthesiologist should always assess the airway for stenosis or other problems, no matter how urgent the situation is.

In this case, a detailed postoperative history revealed that the patient underwent an emergency cesarean section under general anesthesia at our hospital 40 years ago. However, no records were obtained and the patient's memory of the anesthesia procedure was vague, making it difficult to obtain this information during the preoperative medical history interview. Furthermore, the patient underwent knee joint replacement surgery under general anesthesia at our hospital. Nonetheless, no evidence of airway complications was detected in the anesthesia records for this procedure, probably because the airway was secured by LMA.

In this case, the patient was scheduled for intramedullary nail fixation for a humeral fracture in the beach chair position. Furthermore, the patient was obese (body mass index, 36 kg/m^2^); therefore, intubation was considered a better option to secure the airway. Intubation using a smaller tube may be an alternative in cases with SGS. However, as the diameter of the tracheal stenosis site was unknown at the time of SGS discovery, the probability of successful intubation with reduced tube size was not known. Furthermore, we did not want to induce laryngeal edema by repeatedly irritating the glottis. Therefore, with the cooperation of the surgeon, we decided to administer anesthesia using LMA instead of intubation, and the patient's airway was secured without any further complications. Success in this case was the decision to secure the airway with SGA instead of intubation to avoid airway edema once the SGS was identified. Had the LMA not been successful in securing the airway, surgical airway securement, such as cricothyrotomy and incision, would have been considered as needed.

## Conclusions

Here, we report a case of asymptomatic SGS not predicted by preoperative evaluation and discovered during anesthesia induction. The anesthesiologist must be aware that there are cases of asymptomatic patients complicated by SGS.

The LMA is an effective tool for airway management strategies in cases with unexpected intubation complications, including asymptomatic SGS.
